# Remarks upon the Presence of Some Infusoria in the Tissues and Secretions of Patients Dying of Cholera

**Published:** 1850-02

**Authors:** 


*Remarks upon the Presence of some Infusoria in the Tissues and Secretions of patients dying of Cholera.* By W. I. Burnett, M. D.

In the *Am. Jour. of Med. Sci.* for July, 1849, may be found an abstract of a paper of mine, upon the presence of certain *vibriones* in the secretions and tissues of patients dying of cholera.

These observations were made upon some of the first cases of that disease, at its appearance early last summer.

Although these observations were quite limited, it was then thought best to publish immediately the results, as it might serve to direct the attention of observers to this interesting subject.

The facts, therefore, were alone stated, without any pre-judgment of theories.

Since that time, and during the past summer, when the cholera raged so severely in this city, I have had ample opportunities for the pursuance of this subject, in a manner to allow me to speak of it in a general way.

It may be proper to allude first to the cavil of some who have supposed that the whole was an optical illusion, and that the particles in question are only the Brownian molecules in active motion.

This objection belongs only to those who are unacquainted with observations of this kind; and, although the Brownian motion pervades nearly every field of view, yet the adept becomes so accustomed to it, that it can scarce ever be mistaken for that irregular, and apparently voluntary motion belonging to the minute infusoria, and serving as the chief feature of their *animal* nature.

The relations of this infusoria to this disease have many points of interest to be considered.

In almost any rice-water dejection, or the urine of a cholera patient, there may be found millions of these vibriones (*Vibrio prolifer*, Ehren), in active motion; also they are occasionally present in some of the tissues, and especially the muscular, a few hours after the death of the patient; but this last is very far from being common; and I remember to have seen them in these localities in two cases only.

The whole result of my observations upon nearly forty subjects is, that these infusoria are nearly always present in the dejections and urine of those patients, and but rarely so, at least as far as could be seen in the solid tissues.

---

*Report of the Trustees of the Massachusetts General Hospital for 1848, p. 5.

Now, the fact of the presence of the vibriones (the same species), upon the incrustations of teeth, and in diarrhœal dejections from the alimentary canal, has for some time been known to microscopists; and some observations of my own in this direction, during the past summer and autumn, have convinced me that their presence is so common in these situations that it is rare to find an individual in whom they cannot be found; and not only is this species found, but other genera, and especially have I seen a species of the genus Spirillnm, as quite common, not to mention the representatives of other dissimilar genera, which occasionally, though rarely, pass over the field of observation.

All these are merely the inhabitants of the decomposing animal and vegetable matters in which they are found, and, as far as we can learn, occupy a no nearer relation to the system than as being tenants of effete matter, of which the latter is accidentally the depository.

To those acquainted with these facts, the discovery of these infusoria in the tissues and dejections of cholera patients cannot be received with much novelty, or as a result which could not justly have been expected.

Some observations of my own, instituted for the purpose, have shown to me that their presence is perhaps *as* frequently met with in the diarrhœal dejections and tissues of patients dying of other diseases at the same time. And I presume that the vast numbers seen in cholera patients are due to the peculiarity of its symptoms; the vast quantity of water and serum of the blood, continually in the alimentary canal, forms an excellent nidus for their presence and propagation. As to their presence in the tissues, it can only be regarded, as far as I am aware, as closely connected with their incipient decomposition—although this last may be scarcely perceptible to our gross examinations.

Even putting the matter upon still wider grounds, and supposing, for a moment, that these infusoria are to be found only in the dejections and tissues of cholera patients, yet we should then be very far from any evidence which would justly lead us to infer, in the least, their casual relation to this disease.

We know but little of the real relations that the great class of animal parasites hold to the animal economy; and, however close, in any given instance, may be their apparent connection, yet any direct morbific influence on their part can rarely be traced.

All the infusorial parasites of man, as far as I am acquainted (and the infusoria alone can have a bearing on the animalcular theory of any disease), are mere scavengers of the effete particles of the economy.

We are, therefore, induced to allow the famous *Linnæan theory* to rest as an incident of the days in which it was developed.

Since the publication of the first article in the July number of this Journal, there have appeared, in the other journals of this country, several articles upon this subject; but, as they appear to be, for the most part, only confirmations of what was first known, viz., the presence of these infusoria in cholera dejections, I shall pass them over, and briefly allude to some recent articles in the various British journals, which have caught the public eye, as of more import.

The articles to which I particularly refer are those of Dr. Brittan, Mr. Grove, Mr. Basham, and Dr. Swayne—to be found in the last numbers of the *London Lancet, Medical Gazette, Monthly Journal and Retrospect of*

*Medical Sciences,* containing accounts of certain "fungus-like organisms" found in the dejections of cholera patients, and which, in their eyes, have importance and peculiarities sufficient to entitle them to the names of "cholera fungus," and "cholera sporules."

It would be improper for me to speak of these discoveries with the same tone as those of the infusoria, and *their* relations, as I, perhaps, do not precisely know the organisms here spoken of. But I cannot well desist briefly speaking of the matter in a general manner.

Any person at all accustomed to the microscopical examination of the liquid dejections, not only of cholera, but of other patients, must have often been struck with the variety of objects passing over the field of view—epithelial cells so enlarged, and changed by the intestinal contents, as scarcely to be recognized. Also other cells of both an animal and vegetable nature, the germs of which were, perhaps, introduced in food and drink. Besides these, and quite common, is the *Torula crevisiæ,* or yeast plant; and perhaps not quite so frequently as the fungi, as some of the *Uredines.* Dr. Farre (*Trans. of the London Microscop. Soc.,* vol. i. 1844–45) has mentioned an *Alga,* and probably one of the *Occillatoriæ,* as being found in the mucus of the alimentary canal; and Gruby, Lebert, Hannover, and Vogel have described, of late years, the different species from some of the lowest genera of the cryptogamia, which were found in diffetent portions of the alimentary canal, both in health and disease.

These facts being known it is clearly indicated that microscopists cannot be too careful as to the conclusions at which they may arrive, from examinations in this direction.

A continuance of the presence of any of these cryptogamia, in the dejections of patients affected with any epidemic disease, is, of course, a matter not easy of explanation; but, before any deduction can be made, it must first be shown that it is peculiar to this or that affection alone; and, by way of conclusion, and without any pre-judgment of questions, I will say that it cannot appear *very* strange if the "cholera fungus" and "cholera sporules" should ultimately prove to be either new, or some well-known species of the cryptogamia, somewhat modified by circumstances, and which have no higher claim to the humble situation that they occupy, and no nearer relation to the health or disease of the economy, than the "famous vibriones," which we have seen to be the *accidental* tenants of effete matter.

BOSTON, December 10th, 1849. [*Am. Jour. of Med. Sciences.*

